# Differential Expression Profiles and Functional Prediction of Circular RNAs and Long Non-coding RNAs in the Hippocampus of Nrf2-Knockout Mice

**DOI:** 10.3389/fnmol.2019.00196

**Published:** 2019-08-09

**Authors:** Run-Jiao Zhang, Yan Li, Qing Liu, Yan-Jing Gao, Juan Du, Jun Ma, Shao-Guang Sun, Lei Wang

**Affiliations:** ^1^Department of Human Anatomy, Hebei Medical University, Shijiazhuang, China; ^2^School of Nursing, Hebei Medical University, Shijiazhuang, China; ^3^Department of Biochemistry and Molecular Biology, Hebei Medical University, Shijiazhuang, China

**Keywords:** circular RNA, long non-coding RNA, Nrf2, microarray, neuroprotection

## Abstract

**Background:**

Nrf2 (nuclear factor, erythroid 2 like 2) is believed to play a major role in neurodegenerative diseases. The present study attempts to investigate the hippocampal circRNA and lncRNA expression profiles associated with Nrf2-mediated neuroprotection.

**Methods:**

The hippocampal mRNA, circRNA and lncRNA expression profiles of Nrf2 (−/−) mice were determined by a microarray analysis. Bioinformatics analyses, including identification of differentially expressed mRNAs (DEmRNAs), circRNAs (DEcircRNAs) and lncRNAs (DElncRNAs), DEcircRNA-miRNA-DEmRNA interaction network construction, DElncRNA-DEmRNA co-expression network construction, and biological function annotation, were conducted. Quantitative real-time polymerase chain reaction (qRT-PCR) was performed to validate the dysregulated expression of circRNAs and lncRNAs derived from the microarray data of the hippocampus of Nrf2 (−/−) mice.

**Results:**

Compared to wild-type Nrf2 (+/+) mice, 412 DEmRNAs (109 up- and 303 down-regulated mRNAs), 1279 DEcircRNAs (632 up- and 647 down-regulated circRNAs), and 303 DElncRNAs (50 up- and 253 down-regulated lncRNAs) were identified in the hippocampus of Nrf2 (−/−) mice. Additionally, in the qRT-PCR validation results, the expression patterns of selected DEcircRNAs and DElncRNAs were generally consistent with results in the microarray data. The DEcircRNA-miRNA-DEmRNA interaction networks revealed that mmu_circRNA_44531, mmu_circRNA_34132, mmu_circRNA_000903, mmu_circRNA_018676, mmu_circRNA_45901, mmu_circRNA_33836, mmu_circRNA_ 34137, mmu_circRNA_34106, mmu_circRNA_008691, and mmu_circRNA_003237 were predicted to compete with 47, 54, 45, 57, 63, 81, 121, 85, 181, and 43 DEmRNAs, respectively. ENSMUST00000125413, NR_028123, uc008nfy.1, AK076764, AK142725, AK080547, and AK035903 were co-expressed with 178, 89, 149, 179, 142, 55, and 112 DEmRNAs in the Nrf2 (−/−) hippocampus, respectively.

**Conclusion:**

Our study might contribute to exploring the key circRNAs and lncRNAs associated with Nrf2-mediated neuroprotection.

## Introduction

As a transcription factor, Nrf2 (nuclear factor, erythroid 2 like 2) regulates an inducible defense system against oxidative stress and is a member of a small family of basic leucine zipper (bZIP) proteins. After binding to a short antioxidant response element (ARE) in the promoters, Nrf2 triggers the expression of detoxification genes including those involved in redox homeostasis, glutathione turnover, and iron metabolism (Hayes and Dinkova-Kostova, 2014). In the central nervous system (CNS), Nrf2 defends against oxidative stress to provide a protective response by being released from Kelch ECH associating protein 1 (Keap1) and translocating to the nucleus, where it binds the ARE and drives gene expression (Zhang and Hannink, 2003). This protein can prevent oxidant injury in neurons, attenuate NO-dependent neuronal apoptosis, decrease neuronal injury during cerebral ischemia, and ameliorate neurodegeneration in neurodegenerative disease (Shih et al., 2003, 2005; Vargas et al., 2006).

Many neurodegenerative diseases are associated with the failure of specific populations of neurons, such as Parkinson’s disease (PD), Alzheimer’s disease (AD), Huntington’s disease (HD), amyotrophic lateral sclerosis (ALS), and multiple sclerosis (Liddell, 2017). These neurodegenerative diseases share common pathogenic processes, such as oxidative stress, excitotoxicity and glial activation, in which astrocytes gain a reactive phenotype that can be either protective or detrimental to neurons (Sofroniew and Vinters, 2010; Liu B. et al., 2017). Previous studies regarding gain and loss of function of Nrf2 in the brain have suggested that the induction of Nrf2 can ameliorate neurodegeneration, whereas Nrf2 deficiency exacerbates neurodegenerative phenotypes (Zhang et al., 2013). Additionally, Nrf2 is activated in all these neurodegenerative diseases, and Nrf2 activity varies in the regions associated with Alzheimer’s disease, with a tendency for greater Nrf2 activity in the hippocampus and less activity in the frontal cortex (Joshi et al., 2015; Liddell, 2017). Hence, Nrf2 is believed to play a major role in neurodegenerative diseases.

With advances in sequencing technology, various non-coding RNAs have been shown to have significant functions in regulating gene expression (Thai et al., 2013). Circular RNAs (circRNAs), a novel type of endogenous non-coding RNA, are expressed with tissue/developmental stage-specificity and are significantly enriched in the brain (You et al., 2015). An increasing body of evidence indicates the strong association of circRNAs with the development of several CNS diseases, such as AD, PD, and stroke (Floris et al., 2017; Mehta et al., 2017). Additionally, the specific expression of circRNAs and their stability indicate their potential as molecular diagnostic biomarkers for neurodegenerative diseases, such as CDR1 in AD (Lukiw, 2013).

Similarly, as a novel class of RNA transcripts, long non-coding RNAs (lncRNAs) have been demonstrated to be involved in a wide range of epigenetic regulatory mechanisms *via* their direct or indirect interactions with chromatin, and play a critical role in development, differentiation, and homeostasis (Wang and Chang, 2011; Rinn and Chang, 2012). Several studies have explored the relationship between lncRNAs and Nrf2-mediated diseases. Zhang et al. (2015) suggested that low lncRNA Hox transcript antisense intergenic RNA (HOTAIR) expression was associated with down-regulation of Nrf2 in the spermatozoa of patients with asthenozoospermia or oligoasthenozoospermia. Wang et al. (2017) demonstrated that the functional interaction between lncRNA-MEG3 and Nrf2 constitutes the mechanism by which TGF-β2 induces Tenon’s capsule fibroblast proliferation after glaucoma filtration surgery *via* the direct binding of MEG3 to Nrf2. Although the role of circRNAs and lncRNAs in the context of various neurodegenerative diseases has been proposed, the precise mechanism is yet to be elucidated.

In our previous study, we detected the altered expression of circRNAs and lncRNAs in the substantia nigra and corpus striatum tissue of Nrf2-null mice (Liu J. et al., 2017; Yang et al., 2018), and considering that Nrf2 activity may diverge across different brain regions, the current study focused on the expression profiles of circRNAs and lncRNAs in the hippocampus tissue of Nrf2-null mice. Previous studies showed definitively that the hippocampus and neighboring structures of the medial temporal lobe are essential to memory (Preston and Eichenbaum, 2013). The hippocampus plays a critical role in memory formation, which is widely regarded as being central in a brain network that supports encoding and consolidation of memory and, being central to the study of human memory, and has been implicated in episodic and semantic long-term memory (Bartsch and Wulff, 2015; Knierim, 2015). Hence, we further analyzed differentially expressed mRNAs (DEmRNAs), circRNAs (DEcircRNAs) and lncRNAs (DElncRNAs) in the hippocampus between Nrf2 (−/−) and Nrf2 (+/+) mice by microarray analysis. The construction of the DEcircRNA-miRNA-DEmRNA interaction network and DElncRNA-DEmRNA co-expression network was then performed to explore the key circRNAs and lncRNAs correlated with Nrf2-mediated neuroprotection.

## Materials and Methods

### Nrf2-Knockout Mice and Ethics

Three adult male Nrf2 (+/+) mice (25 to 30 g, 3 to 4 months, *n* = 3) and three Nrf2 (−/−) mice (25 to 30 g, 3 to 4 months, *n* = 3), which were kindly provided by academician Chun-Yan Li (Department of Neurology, Second Hospital of Hebei Medical University, Shijiazhuang, China), were used for this study. The present study utilized mice with an ICR (Institute of Cancer Research) background. Three mice were randomly selected for each group (*n* > 20) in a random manner. The mice were housed in a 12 h light/dark cycle in a temperature-controlled environment and fed *ad libitum*, with 4 to 5 mice per cage. None of these mice underwent perfusion. By using polymerase chain reaction (PCR) amplification of genomic DNA from tails, the genotypes [(Nrf2 (+/+) and Nrf2 (−/−)] of the mice were determined. The PCR primers used in the identification of genotypes and the electrophoresis image are shown in Supplementary Figure S1 and Supplementary Table S1, respectively. All the mice were sacrificed by using an overdose of an isoflurane/oxygen mixture (Huazhong Haiwei Gene Technology, Co., Ltd., Cat. No. 021400, Beijing, China). Hippocampal tissues of each mouse were obtained from surgery, and immediately homogenized for the extraction of total RNA (TRIzol, Invitrogen, Cat No. 15596026, Carlsbad, CA, United States).

In this study, the animal experiments complied with the regulations of the Animal Welfare Act of the National Institutes of Health Guide for the Care and Use of Laboratory Animals (NIH Publication No. 85-23, revised 1996) and were approved by the ethics committee of Hebei Medical University (IACUC-Hebmu-Glp-2016017). All investigators were blinded. The study was not pre-registered.

### CircRNA Microarrays

The circRNA microarray was analyzed using Arraystar Mouse circRNA Array V2 analysis (Arraystar, Inc., United States) by Kangchen BioTech, Inc. (Shanghai, China). By using a NanoDrop ND-1000 instrument (Nanodrop Technologies, Inc.), the concentration of total RNA was determined by OD260. The integrity of total RNA was assessed by electrophoresis (Liuyi Instrument Factory, Cat No. DDY-5, Beijing, China) on a denaturing agarose gel (Gene Company, Ltd., Cat No. EEO015, China).

### LncRNA and mRNA Microarrays

Arraystar Mouse LncRNA Microarray V3.0 (Rockville, MA, United States) is designed for the global profiling of mouse lncRNAs and protein-coding transcripts. The lncRNAs were carefully constructed using the most reputed public transcription databases (Refseq, UCSC known genes, Ensembl, etc.), as well as landmark publications. Each transcript is represented by a specific exon or splice junction probe, which can accurately identify the individual transcripts. Positive probes for housekeeping genes and negative probes were also printed onto the array for hybridization quality control.

### RNA Labeling and Array Hybridization

Based on the manufacturer’s protocol, sample-circRNA labeling and array hybridization were performed. Briefly, to remove linear RNAs and enrich circular RNAs, total RNA was digested with RNase R (Epicentre, Inc., Cat No. RNR07250, United States) and the enriched circular RNAs were amplified and transcribed into fluorescent cRNA by utilizing a random priming method with an Arraystar Super RNA Labeling Kit (Arraystar, Inc., Cat No. AS-LE-005, United States). With a RNeasy Mini Kit (Qiagen, Cat No. 74104, Germany), the fluorophore-labeled cRNA was purified. By using a NanoDrop ND-1000 (Nanodrop Technologies, Inc.), the concentration and specific activity of labeled cRNAs (pmol Cy3/μg cRNA) were measured. One microgram of each labeled cRNA was fragmented by adding 5 μl 10× Blocking Agent and 1 μl of 25× Fragmentation Buffer, and then, the mixture was heated at 60°C for 30 min. Finally, 25 μl 2× hybridization buffer (Agilent, Cat No. 5190-0403, United States) was added to dilute the labeled cRNA. Hybridization solution (50 μl) was dispensed into the gasket slide (Agilent, Cat No. G2534-60003, United States) and assembled to a mouse circRNA microarray (8 × 15K, Arraystar) slide. In an Agilent hybridization oven (Agilent, Cat No. G2545A, United States), the slides were incubated for 17 h at 65°C. The hybridized arrays were washed, fixed and scanned using the Agilent Scanner G2505C (Agilent, Cat No. G2565BA, United States) and scanned images were imported into Agilent Feature Extraction software for raw data extraction. Quantile normalization and subsequent data processing were performed by using the Kangchen homemade R software package (Kangchen BioTech, Inc., Shanghai, China), and then, low intensity filtering was performed.

With minor modifications of the Agilent One-Color Microarray-Based Gene Expression Analysis protocol (Agilent Technology, Palo Alto, CA, United States), sample-lncRNA labeling and array hybridization were performed. Briefly, after removal of rRNA, mRNA was purified from total RNA (mRNA-ONLY^TM^ Eukaryotic mRNA Isolation Kit, Epicentre, Madison, WI, United States). Then, along the entire length of the transcripts without a 3′ bias utilizing a random priming method, each sample was amplified and transcribed into fluorescent cRNA (Arraystar Flash RNA Labeling Kit, Arraystar, Rockville, MA, United States). By using a RNeasy Mini Kit (Qiagen, Valencia, CA, United States), the labeled cRNAs were purified. The concentration and specific activity of the labeled cRNAs (pmol Cy3/μg cRNA) were measured by NanoDrop ND-1000 (NanoDrop Technologies, Thermo Scientific, Wilmington, DE, United States). By adding 5 μl 10× Blocking Agent and 1 μl of 25× fragmentation buffer, 1 μg of each labeled cRNA was fragmented, and the mixture was heated at 60°C for 30 min. Finally, 25 μl 2× GE Hybridization buffer was added to dilute the labeled cRNA. Hybridization solution (50 μl) was dispensed into the gasket slide (Agilent, Cat No. G2534-60003, United States) and assembled to the lncRNA expression microarray slide. The slides were incubated for 17 h at 65°C in an Agilent Hybridization Oven (Agilent, Cat No. G2545A, United States). The hybridized arrays were washed, fixed and scanned with the Agilent DNA Microarray Scanner (part number G2505C).

### Data Pre-processing

To analyze the acquired array images, Agilent Feature Extraction software (version 11.0.1.1) was used. Quantile normalization and subsequent data processing were performed using the GeneSpring GX v12.1 software package (Agilent Technologies). After quantile normalization of the raw data, lncRNAs and mRNAs with flags of at least three out of six samples were chosen for further data analysis. Through *p*-value filtering, lncRNAs and mRNAs with statistical significance between the two groups were identified. DElncRNAs and DEmRNAs between the two samples were identified through fold change filtering. By using homemade scripts, hierarchical clustering, and combined analysis were performed.

### DEmRNA, DEcircRNA, and DElncRNA Analyses

The expression profiles of mRNAs, circRNAs, and lncRNAs in the hippocampal tissues of Nrf2 (+/+) and Nrf2 (−/−) mice were obtained. The calculation of fold change (FC) and *p*-value between these groups for each mRNA, circRNA, and lncRNA was performed to compare the profile differences in the hippocampal tissues between Nrf2 (+/+) and Nrf2 (−/−) mice. By using a *t*-test, the statistical significance of the difference was estimated. While mRNAs and circRNAs with FC > 1.5 and *p*-values < 0.05 were selected as the significantly DEcircRNAs and DEmRNAs, lncRNAs with *p* < 0.05 and FC > 2 were identified as DElncRNAs. By using the ‘pheatmap’ package in R language, hierarchical cluster analysis of group samples based on expression values of mRNAs, circRNAs, and lncRNAs was conducted.

### Quantitative Real-Time Polymerase Chain Reaction (qRT-PCR) Validation

According to the manual instructions, total RNA of the hippocampal tissues generated from Nrf2 (−/−) mice and Nrf2 (+/+) mice was extracted using TRIzol (Thermo Fisher Scientific, Wilmington, DE, United States). By using an M-MLV First Strand Kit (Thermo Fisher Scientific, Inc., Cat No. 00341186, United States), total RNA (2 μg) was transcribed to cDNA. By using SuperReal PreMix Plus (SYBR Green) (TIANGEN, Cat No. FP205, China) with an Illumina Eco PCR machine (Illumina, San Diego, CA, United States), qRT-PCR was performed. QRT-PCR reaction conditions were as follows: an initial denaturation step of 15 min at 95°C, followed by 40 cycles of 15 s at 95°C and 20 s at 55°C, 20 s at 72°C. For normalization, β-actin was selected as the housekeeping gene. All experiments were performed in triplicate. The 2^–Δ^ ^Δ^ ^*CT*^ method was used to calculate relative fold changes for quantitative results. The PCR primers are displayed in Table 1.

**TABLE 1:** The primers used in qRT-PCR experiments.

**circRNA**	**Primers**
mmu_circRNA_44531	Forward: 5′ TGAATGGCTCTCACCCTGTCTCCTT 3′
	Reverse: 5′ GCAGTGGCGGACACAGATCTTTAAG 3′
mmu_circRNA_34132	Forward: 5′ TGAAGAACTCTGTCTACCGAAGCC 3′
	Reverse: 5′ TAGTCAAAGCCTTGCACGGGAT 3′
mmu_circRNA_000903	Forward: 5′ ACATGCTCCAGGCCCCTCTGTTTAC 3′
	Reverse: 5′ GATGAGTGCTTTGCGGAATTTGGTG 3′
mmu_circRNA_018676	Forward: 5′ ACACCCTTTATGATGTCACACCTTT 3′
	Reverse: 5′ TCCAAAAGAATAGGCTGGCTGAG 3′
mmu_circRNA_45901	Forward: 5′ TCAGGCTCGGCTACAGAAGGAAC 3′
	Reverse: 5’ ATAAGATATTGCCACGGTGGATGAA 3′
mmu_circRNA_33836	Forward: 5′ GAACCTTACTCAAAAGCATCCCACT 3′
	Reverse: 5’ TTGGGCCAACATGTATTATTCTTCC 3′
mmu_circRNA_34137	Forward: 5′ TCAGCACCAGGAGCAACCTAACAG 3′
	Reverse: 5′ TCTTGAATCTGTCTGCTGCTGAGGA 3′
mmu_circRNA_34106	Forward: 5′ GGCTGCTGAAGAGTGAACTTGGAT 3′
	Reverse: 5′ AGGTGAGGATGGAGCTGTGTCTTC 3′
mmu_circRNA_008691	Forward: 5′ACACTCATTATTGGCTGGGGAACTT 3′
	Reverse: 5′ CACACTCCTTATGTTTCCTTCCCCT 3′
mmu_circRNA_003237	Forward: 5′ AGACGTGGAACCTGCTCAATC 3′
	Reverse: 5′ GACCTTCCCCAAAACAGACTTC 3′
**lncRNA**	**Primers**
uc008nfy.1	Forward: 5′ ATGTCAGGCAGCAGGGACC 3′
	Reverse: 5′ TCCTTGCTTCCTCTTTGGGTT 3′
ENSMUST00000125413	Forward: 5′ TCCCTGAGTGTTTATATTTACAGATGGC 3′
	Reverse: 5′ GCCACACAGTAGGAAGATGAGACCA 3′
NR_028123	Forward: 5′ GCTCTTCCCCAGTTTCAAGTTCTCA 3′
	Reverse: 5′ CATTCCAGTCCTCAGATACGCTCAG 3′
AK076764	Forward: 5′ TATGGACGATATTGTGATTTGTGGACC 3′
	Reverse: 5′ AGTGGCTCCTAAAAAATGACTAACGTC 3′
AK142725	Forward: 5′ AAGGAGTGAAGCCACAGCA 3′
	Reverse: 5′ CCTCAGACACAAGAGAAACCAG 3′
AK080547	Forward: 5′ TGAGTGTTGTGAGTCTAGGTGAGCT 3′
	Reverse: 5′ TCCTTGCCAGAGTCTAATGATACCT 3′
AK035903	Forward: 5′ ACGGCACCTTTATCCAGCCATCT 3′
	Reverse: 5′ TCTGGAGGGTCTGAAGACAGCTACA 3′
b-Actin	Forward: 5′ TCATCACTATTGGCAACGAGCGGT 3′
	Reverse: 5′ GTGTTGGCATAGAGGTCTTTACG 3′

*qRT-PCR, quantitative real-time polymerase chain reaction; circRNA, circular RNA; lncRNA, long non-coding RNA.*

### Functional Annotation of DEmRNAs

Gene Ontology (GO) classification and the Kyoto Encyclopedia of Genes and Genomes (KEGG) pathway enrichment were conducted using the clusterProfiler of R/Bioconductor^1^. According to the size of the enrichment factor, we extracted the top 30 terms/pathways.

### Construction of DEcircRNA-miRNA-DEmRNA Interaction Networks

The DEcircRNA-miRNA-mRNA interactions were predicted with Arraystar’s homemade miRNA target prediction software based on TargetScan & MiRanda (Enright et al., 2003; Pasquinelli, 2012). The miRNA–mRNA interactions with total context + + ≤ −0.2 were selected for further analysis. In these DEcircRNA-miRNA-mRNA interaction networks, the circRNAs and mRNAs were linked by shared miRNAs predicted as downstream targets of circRNAs and upstream regulators of mRNAs. Thus, the change in circRNA and mRNA should be in the same direction, and then, we selected DEmRNAs that were indirectly positively regulated by DEcircRNAs for the following research. The DEcircRNA-miRNA-DEmRNA interaction networks were visualized using Cytoscape (version 3.7.0)^2^. GO classification and KEGG pathway enrichment were conducted for the target DEmRNAs of miRNAs sponged by DEcircRNAs in the hippocampal tissues using the clusterProfiler of R/Bioconductor^1^. According to the size of the enrichment factor, we extracted the top 10 terms/pathways.

### Construction of DElncRNA-DEmRNA Co-expression Networks

According to their expression levels, the Pearson correlation coefficient (PCC) was used to depict the co-expression relationship between DElncRNAs and DEmRNAs. DElncRNA-DEmRNA pairs with | PCC| > 0.90 and *p* < 0.05 were retained for network construction, which was deciphered by Cytoscape 3.1^2^. GO classification and KEGG pathway enrichment for DEmRNAs co-expression with DElncRNAs were conducted using the clusterProfiler of R/Bioconductor^1^. According to the size of the enrichment factor, we extracted the top 10 terms/pathways.

### Statistical Analysis

The investigators were blinded to animal groups to reduce experimenter bias and to achieve unbiased results. No sample size calculation was performed. There were no sample size differences between the beginning and end of the experiments. And qRT-PCR was performed in triplicate. Functional annotation was conducted using the clusterProfiler of R/Bioconductor^1^. Mean ± standard deviation and independent-samples *t*-tests were used in the statistical analysis based on Agilent Genespring GX Linux software (Version 11.5) and IBM SPSS Statistics software (Version 22.0), respectively. Outliers were deleted. A value of *p* < 0.05 was considered the criterion of statistical significance.

## Results

### DEmRNAs Between the Nrf2 (−/−) Mice and Nrf2 (+/+) Mice

**FIGURE 1:**
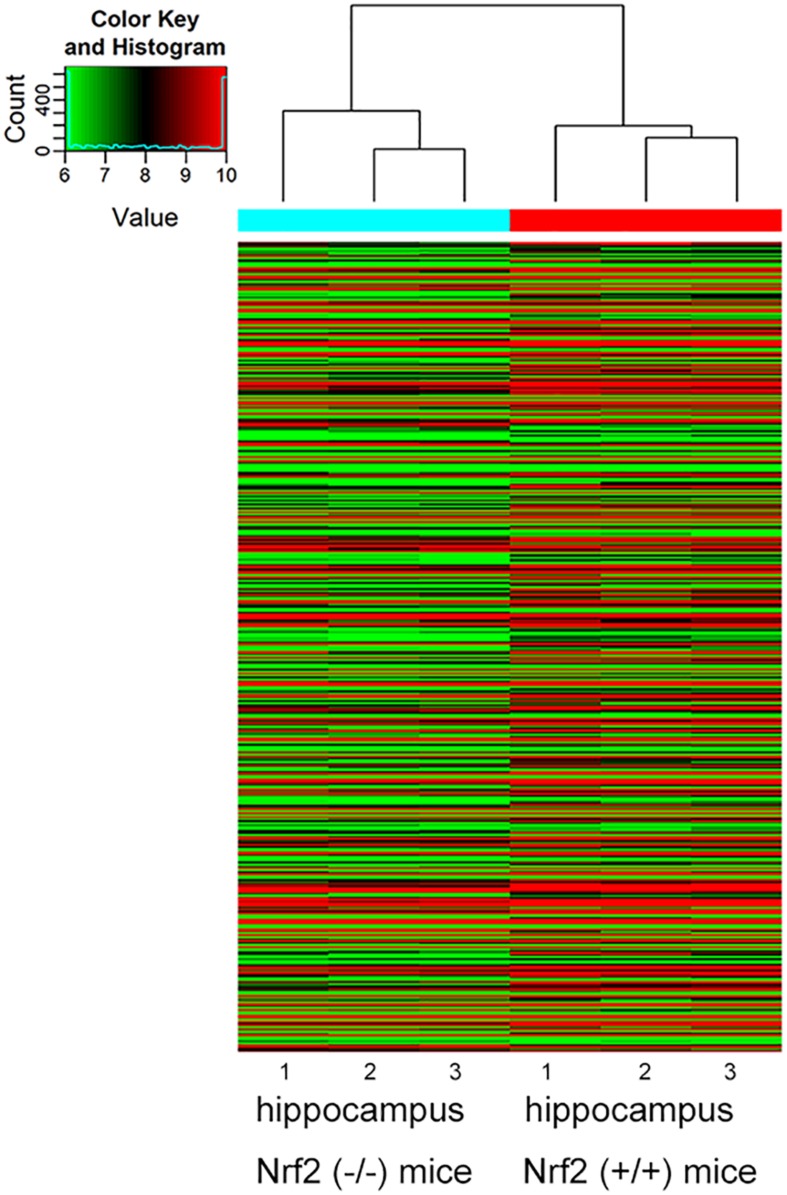
Hierarchical clustering analysis based on the expression profile of the DEmRNAs in the Nrf2 (–/–) hippocampus. *n* = 3.

**FIGURE 2:**
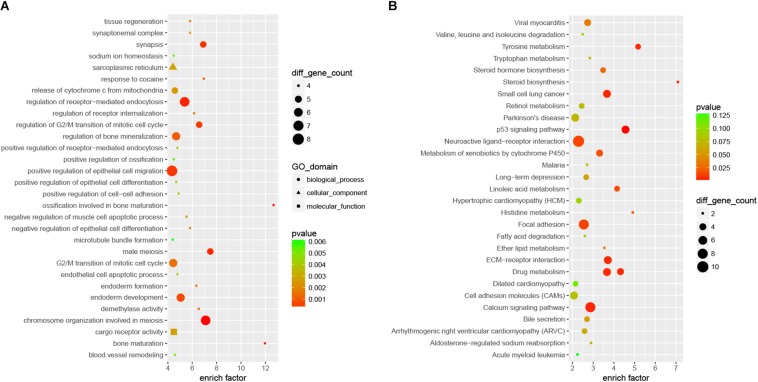
The top 30 most significantly enriched GO **(A)** and KEGG **(B)** pathways of DEmRNAs between the Nrf2 (–/–) and Nrf2 (+/+) hippocampus. The x-axis shows counts of genes enriched in GO and KEGG pathways, and the y-axis shows the GO and KEGG pathways. The color scale depicts the *p*-value.

Compared with Nrf2 (+/+) mice, a total of 412 DEmRNAs, including 109 up-regulated and 303 down-regulated mRNAs, were detected in the hippocampus with a >1.5-fold change and *p*-value < 0.05 (Figure 1 and Supplementary Table S2). Functional annotation of DEmRNAs in the hippocampal tissues was conducted. GO enrichment analysis (Figure 2A) indicated that, the DEmRNAs were enriched in response to cocaine (biological process: 0042220, *p* = 9.471e-04), synapsis (biological process: 0007129, *p* = 3.246e-04), demethylase activity (molecular function: 0032451, *p* = 1.224e-03), synaptonemal complex (cellular component: 0000795, *p* = 1.952e-03), and release of cytochrome c from mitochondria (biological process: 0001836, *p* = 2.416e-03). KEGG pathway enrichment analysis for significantly dysregulated mRNAs was useful to reveal related pathways and molecular interactions. According to the KEGG enrichment analysis (Figure 2B), these DEmRNAs were enriched in the pathways of the calcium signaling pathway (KEGG: mmu04020, *p* = 5.901e-03), long-term depression (LTD) (KEGG: mmu04730, *p* = 5.908e-02), and Parkinson’s disease (KEGG: mmu05012, *p* = 7.166e-02), which were closely correlated with neurodegenerative diseases.

### DEcircRNAs and DElncRNAs in the Hippocampus Between the Nrf2 (−/−) Mice and Nrf2 (+/+) Mice

**FIGURE 3:**
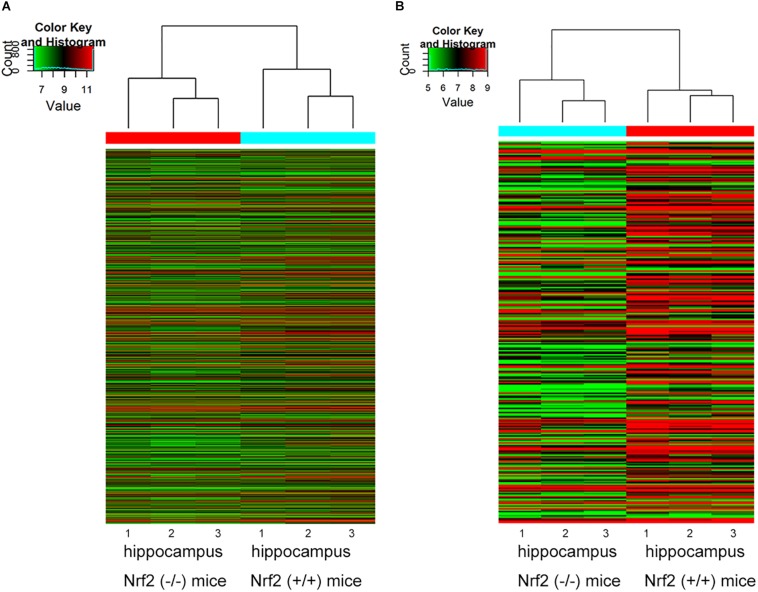
Hierarchical clustering analysis based on the expression profile of the DEcircRNAs **(A)** and DElncRNAs **(B)** in Nrf2 (–/–) the hippocampus. *n* = 3.

Compared to Nrf2 (+/+) mice, a total of 1279 DEcircRNAs, including 632 up-regulated circRNAs and 647 down-regulated circRNAs, were detected in the hippocampus between Nrf2 (−/−) and Nrf2 (+/+) mice with a >1.5-fold change and *p*-value < 0.05 (Supplementary Tables S3, S4). Among them, mmu_circRNA_40139 and mmu_circRNA_26611 were the most up-regulated and down-regulated DEcircRNAs in the hippocampus, respectively (Table 2). A total of 303 DElncRNAs, including 50 up-regulated and 253 down-regulated lncRNAs, were identified in the hippocampus of Nrf2 (−/−) mice with a >2-fold change and *p*-value < 0.05. ENSMUST00000161755 and ENSMUST00000105610 were the most up-regulated and down-regulated DElncRNAs in the hippocampus, respectively (Table 3). Hierarchical clustering of these DEcircRNAs and DElncRNAs indicated an obvious discrimination between Nrf2 (−/−) and Nrf2 (+/+) mice (Figure 3). The raw-data have been uploaded to Gene Expression Omnibus (GEO) (GSE122421, https://www.ncbi.nlm.nih.gov/geo/query/acc.cgi?acc=GSE122421; GSE122422, https://www.ncbi.nlm.nih.gov/geo/query/acc.cgi?acc=GSE122422).

**TABLE 2:** Top 10 up- and down-regulated DEcircRNAs in the hippocampus of Nrf2 (−/−) mice.

**circRNA**	**circRNA_ type**	**chrom**	***p*-value**	**Regulation**
mmu_circRNA_40139	Exonic	chr6	0.00005436006	Up
mmu_circRNA_44715	Exonic	chr9	0.000203188	Up
mmu_circRNA_27509	Exonic	chr14	0.000218135	Up
mmu_circRNA_34109	Sense overlapping	chr2	0.000227424	Up
mmu_circRNA_28600	Exonic	chr15	0.000264287	Up
mmu_circRNA_33745	Sense overlapping	chr2	0.000283733	Up
mmu_circRNA_26322	Exonic	chr13	0.000325369	Up
mmu_circRNA_29992	Exonic	chr16	0.000380296	Up
mmu_circRNA_34863	Exonic	chr2	0.000393293	Up
mmu_circRNA_33826	Exonic	chr2	0.000403672	Up
mmu_circRNA_26611	Sense overlapping	chr13	0.000155304	Down
mmu_circRNA_34832	Exonic	chr2	0.000165934	Down
mmu_circRNA_35957	Exonic	chr3	0.000227463	Down
mmu_circRNA_34137	Exonic	chr2	0.000492821	Down
mmu_circRNA_44589	Exonic	chr9	0.000527078	Down
mmu_circRNA_43020	Exonic	chr8	0.000534644	Down
mmu_circRNA_34314	Exonic	chr2	0.000545661	Down
mmu_circRNA_33836	Exonic	chr2	0.000564463	Down
mmu_circRNA_30123	Exonic	chr16	0.000678283	Down
mmu_circRNA_33850	Exonic	chr2	0.00071285	Down

*DEcircRNAs, differentially expressed circRNAs.*

**TABLE 3:** Top 10 up- and down-regulated DElncRNAs in the hippocampus of Nrf2 (−/−) mice.

**Seqname**	**Gene symbol**	***p*-value**	**FDR**	**Fold change**	**Regulation**
ENSMUST00000161755	Setd7	1.04874E-05	0.090718311	19.0132304	Up
uc012cpj.1	5031434O11Rik	7.82829E-05	0.155417706	8.3313618	Up
AK037363	AK037363	0.000152779	0.204707508	3.5895181	Up
uc008nfy.1	AK006531	0.000668524	0.379212113	2.2272505	Up
AK139259	AK139259	0.000874653	0.432610759	2.789535	Up
AK139259	AK139259	0.000874653	0.432610759	2.789535	Up
ENSMUST00000170420	Dusp12	0.001161771	0.436947138	3.0719131	Up
ENSMUST00000137756	Gm13371	0.001280493	0.436947138	2.4390113	Up
ENSMUST00000143128	Chia	0.001577294	0.436947138	4.6602429	Up
ENSMUST00000139157	Tmeff2	0.001698904	0.437780578	3.5387142	Up
ENSMUST00000105610	Gm17106	1.83126E-05	0.090718311	50.0259527	Down
TCONS_00018071	XLOC_014960	1.84989E-05	0.090718311	12.0584926	Down
NR_028263	B130006D01Rik	3.07853E-05	0.091678721	6.5836299	Down
ENSMUST00000123574	Gm14002	0.000101993	0.186914246	6.3845782	Down
ENSMUST00000123574	Gm14002	0.000101993	0.186914246	6.3845782	Down
ENSMUST00000126340	Dcdc5	0.000162376	0.204707508	14.695374	Down
AK144955	AK144955	0.000197566	0.22130871	7.9976315	Down
NR_003293	D030028A08Rik	0.000200719	0.22130871	10.7616687	Down
NR_003293	D030028A08Rik	0.000200719	0.22130871	10.7616687	Down
AK076764	AK076764	0.000204365	0.22130871	2.1472701	Down

*DElncRNAs, differentially expressed lncRNAs; FDR, false discovery rate.*

### QRT-PCR Validation of the Expression of the Selected DEcircRNAs and DElncRNAs

**FIGURE 4:**
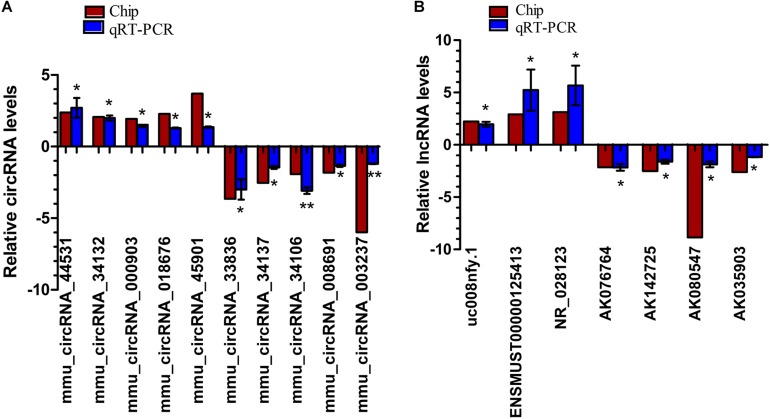
QRT-PCR validation of the expression levels of candidate circRNAs **(A)** and lncRNAs **(B)**. ^*^p < 0.05 and ^∗∗^*p* < 0.01. The deep red column indicates the expression status of lncRNAs through microarray analyses; the blue column indicates the expression status of lncRNAs through qRT-PCR experiments. *n* = 3.

Ten dysregulated circRNAs, including five up-regulated (mmu_circRNA_44531, mmu_circRNA_34132, mmu_circRNA_000903, mmu_circRNA_018676, and mmu _circRNA_45901) and five down-regulated DEcircRNAs (mmu_circRNA_33836, mmu_circRNA_34137, mmu_circRNA_ 34106, mmu_circRNA_008691, and mmu_circRNA_003237), and seven dysregulated lncRNAs, including three up-regulated (ENSMUST00000125413, NR_028123, and uc008nfy.1) and four down-regulated DElncRNAs (AK076764, AK142725, AK080547, and AK035903), with relatively high fold changes, high expression and low *p*-values, were selected for qRT-PCR validation. Compared to Nrf2 (+/+) mice, the 10 dysregulated circRNAs and seven dysregulated lncRNAs were successfully verified by qRT-PCR. Generally, the expression patterns of these selected circRNAs and lncRNAs in qRT-PCR validation results were consistent with those in our microarray results (Figure 4).

### DEcircRNA-miRNA-DEmRNA Interaction Networks

**FIGURE 5:**
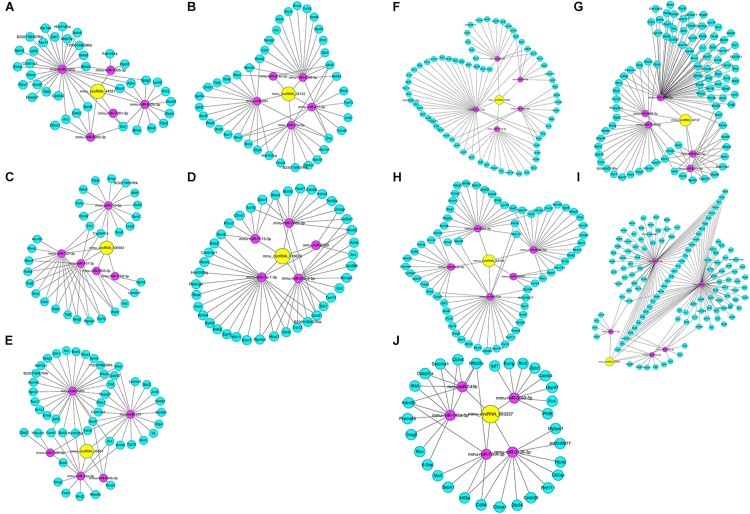
DEcircRNA-miRNA-DEceRNA interaction subnetworks of up-regulated circRNAs and down-regulated circRNAs in the Nrf2 (–/–) hippocampus. **(A)** Subnetwork of mmu_circRNA_44531 in the Nrf2 (–/–) hippocampus. **(B)** Subnetwork of mmu_circRNA_34132 in the Nrf2 (–/–) hippocampus. **(C)** Subnetwork of mmu_circRNA_000903 in the Nrf2 (–/–) hippocampus. **(D)** Subnetwork of mmu_circRNA_018676 in the Nrf2 (–/–) hippocampus. **(E)** Subnetwork of mmu_circRNA_45901 in the Nrf2 (–/–) hippocampus. **(F)** Subnetwork of mmu_circRNA_33836 in the Nrf2 (–/–) hippocampus. **(G)** Subnetwork of mmu_circRNA_34137 in the Nrf2 (–/–) hippocampus. **(H)** Subnetwork of mmu_circRNA_34106 in the Nrf2 (–/–) hippocampus. **(I)** Subnetwork of mmu_circRNA_008691 in the Nrf2 (–/–) hippocampus. **(J)** Subnetwork of mmu_circRNA_003237 in the Nrf2 (–/–) hippocampus. Yellow nodes indicate DEcircRNAs. Magenta and green nodes indicate miRNAs sponged by DEcircRNAs and the gene ID of their DEceRNAs, respectively. Edges represent interactions.

**FIGURE 6:**
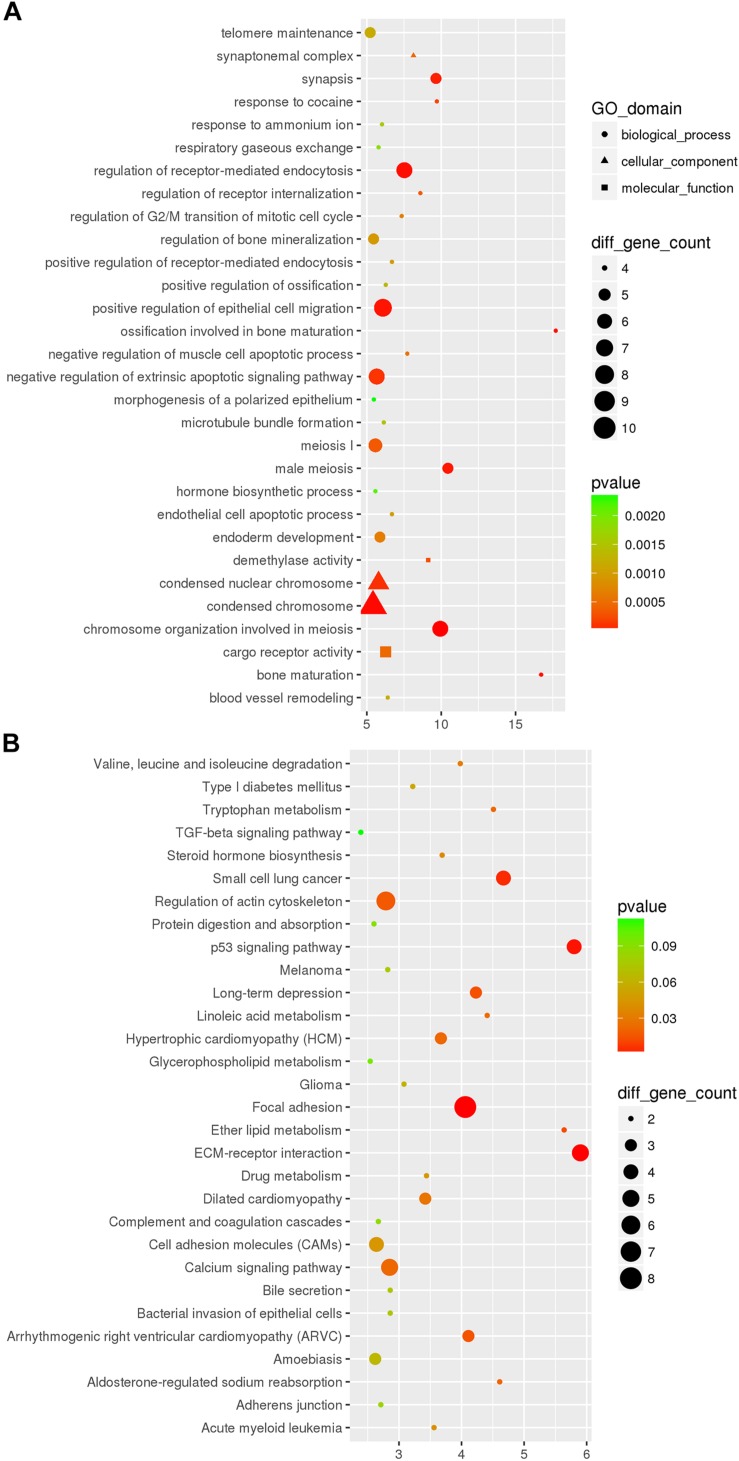
The top 30 most significantly enriched GO **(A)** and KEGG **(B)** pathways of DEceRNAs of miRNAs sponged by DEcircRNAs between the Nrf2 (–/–) and Nrf2 (+/+) hippocampus. The x-axis shows counts of genes enriched in GO **(A)** and KEGG **(B)** pathways and the y-axis shows the GO and KEGG pathways. The color scale depicts the *p*-value.

In the DEcircRNA-miRNA-DEmRNA interaction networks, mmu_circRNA_44531, mmu_circRNA_34132, mmu_circRNA_ 000903, mmu_circRNA_018676, mmu_circRNA_45901, mmu_ circRNA_33836, mmu_circRNA_34137, mmu_circRNA_34106, mmu_circRNA_008691, and mmu_circRNA_003237 were predicted to compete with 47, 54, 45, 57, 63, 81, 121, 85, 181, and 43 DEmRNAs, respectively. The DEcircRNA-miRNA-DEmRNA interaction networks are shown in Figure 5. In the GO enrichment analysis (Figure 6A), DEmRNAs that shared the same binding miRNAs with DEcircRNAs were enriched in chromosome organization involved in meiosis (biological process: 0070192, *p* = 0.00000278), condensed chromosome (cellular component: 0000793, *p* = 0.00001108), negative regulation of extrinsic apoptotic signaling pathway (biological process: 2001237, *p* = 0.00012), response to cocaine (biological process: 0042220, *p* = 0.0002086), demethylase activity (molecular function: 0032451, *p* = 0.0002718), negative regulation of muscle cell apoptotic process (biological process: 0010656, *p* = 0.0005509), and telomere maintenance (biological process: 0000723, *p* = 0.001168). According to the KEGG enrichment analysis (Figure 6B), these DEmRNAs were enriched in the pathways of ECM-receptor interaction (KEGG: mmu04512, *p* = 0.0006341), focal adhesion (KEGG: mmu04510, *p* = 0.000646), LTD (KEGG: mmu04730, *p* = 0.01344), calcium signaling pathway (KEGG: mmu04020, *p* = 0.02193), and type I diabetes mellitus (KEGG: mmu04940, *p* = 0.05376). Three DEmRNAs (IGF1, RYR1, and PLA2G12A) were enriched in LTD. Among them, IGF1 and RYR1 were targets of mmu_circRNA_34106 and mmu_circRNA_008691; RYR1 was the target of mmu_circRNA_34137; and PLA2G12A was the target of mmu_circRNA_33836.

### DElncRNA-DEmRNA Co-expression Networks

**FIGURE 7:**
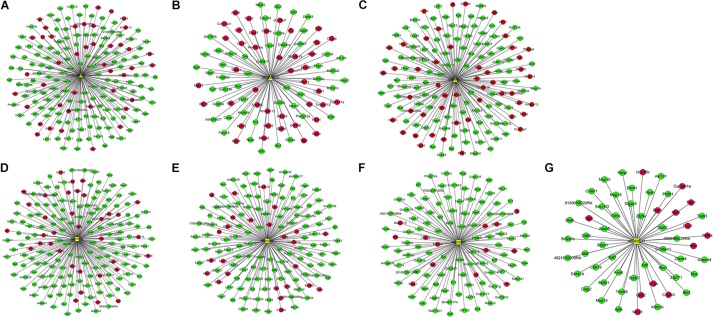
Co-expression network between up- and down-regulated DElncRNAs and DEmRNAs in the Nrf2 (–/–) hippocampus. **(A)** Subnetwork of ENSMUST00000125413. **(B)** Subnetwork of NR_028123. **(C)** Subnetwork of uc008nfy.1. **(D)** Subnetwork of AK076764. **(E)** Subnetwork of AK142725. **(F)** Subnetwork of AK035903. **(G)** Subnetwork of AK080547. The triangles, squares represent up- and down-regulated DElncRNAs, and ellipses represent DEmRNAs in the Nrf2 (–/–) hippocampus. Red and blue color represent up- and down-regulation in the Nrf2 (–/–) hippocampus, respectively.

The DElncRNA-DEmRNA co-expression networks, which included the previously mentioned DEmRNAs co-expressed with seven DElncRNAs, were constructed based on expression profiling. ENSMUST00000125413, NR_028123 and uc008nfy.1, which were up-regulated DElncRNAs in Nrf2 (−/−) hippocampal tissues, co-expressed with 178, 89, and 149 DEmRNAs (Figures 7A–C). AK076764, AK142725, AK080547 and AK035903, which were down-regulated DElncRNAs in Nrf2 (−/−) hippocampal tissues, co-expressed with 179, 142, 55, and 112 DEmRNAs (Figures 7D–G). GO enrichment analysis indicated that, the DEmRNAs co-expressed with DElncRNAs were enriched in the release of sequestered calcium ion into cytosol (biological process: 0051209), regulation of sequestering of calcium ion (biological process: 0051282), and release of cytochrome c from mitochondria (biological process: 0001836). According to the KEGG enrichment analysis, these DEmRNAs co-expressed with DElncRNAs were enriched in the pathways of calcium signaling pathway (KEGG: mmu04020), LTD (KEGG: mmu04730), and Parkinson’s disease (KEGG: mmu05012) (Supplementary Table S5).

## Discussion

In recent years, Nrf2, as a cytoprotective transcription factor with antioxidant effects, has aroused broad interest. Many microarray analyses based on the expression profiling of liver, intestine, and lung tissues of Nrf2 (−/−) mice have been performed (Enomoto et al., 2001; Thimmulappa et al., 2002; Rangasamy et al., 2004). Activation of the Nrf2-ARE pathway has been shown to confer benefits on many neurodegenerative disorders in animal models, which supports the concept of developing pharmaceuticals to activate the Nrf2-ARE pathway in the brain. As an indicator and regulator of oxidative stress, the Nrf2-ARE pathway has been shown to undergo dynamic changes and has been examined for its neuroprotective role in many cases (Li and Johnson, 2014). Nevertheless, further studies on Nrf2 need to be performed. To reveal the important role of circRNAs and lncRNAs associated with Nrf2-mediated neuroprotection in the brain, DEcircRNAs and DElncRNAs in the hippocampal tissues between Nrf2 (−/−) and Nrf2 (+/+) mice were identified by microarray and bioinformatics analysis.

The hippocampus is implicated in episodic memory and plays a pivotal role in particular aspects of the acquisition of semantic or factual knowledge (Eichenbaum, 2004). Long-term memory consists of episodic memory and semantic memory, which is closely associated with LTD and long-term potentiation (LTP). In general, memories are reorganized into widely distributed cortical networks over time through systems-level consolidation. At the cellular level, it is believed that storage of information initially occurs *via* altered synaptic strength through processes such as LTP (Santini et al., 2014). Synaptic plasticity is believed to be a putative biological substrate for learning and memory processes, and a mammalian target of rapamycin (mTOR) contributes to regulating synaptic remodeling and long-term synaptic plasticity in the hippocampus (Tang et al., 2002; Ma et al., 2010; Pinar et al., 2017). LTD and LTP, as two main forms of synaptic plasticity in the brain, have been demonstrated to account for the onset and progression of motor symptoms of PD (Picconi et al., 2012). Plastic changes in synaptic efficacy, such as LTD and LTP, are widely recognized as mechanisms implicated in learning and memory, responses to drugs of abuse, and addiction (Lovinger and Abrahao, 2018). In the CA1 region, a hippocampal subregion, the induction of homosynaptic LTD requires the activation of *N*-methyl-D-aspartate (NMDA) receptors (Dudek and Bear, 1992), voltage-gated calcium channels (Christie et al., 1994, 1997) and/or calcium release from intracellular stores (Nishiyama et al., 2000).

In our analysis, four DEmRNAs (SLC6A3, DRD2, LPHN3, and DLG4) were enriched in response to cocaine, which was associated with addiction. Five DEmRNAs (STAG3, TEX15, CCNB1IP1, MAEL, and TEX11) were enriched in synapses. Five DEmRNAs (TRHR, P2RX1, CAMK4, CACNA1G, and RYR1) were enriched in the calcium signaling pathway. As mentioned above, three DEmRNAs (IGF1, RYR1, and PLA2G12A) were enriched in LTD. Among them, SLC6A3, TRHR, TEX15, MAEL, CACNA1G, and PLA2G12A were targets of mmu_circRNA_33836; SLC6A3, LPHN3, TEX15, MAEL, TRHR, RYR1, and IGF1 were targets of mmu_circRNA_008691; DLG4, STAG3, CCNB1IP1, TEX11, and P2RX1 were targets of mmu_circRNA_45901; and CAMK4, CACNA1G, and RYR1 were targets of mmu_circRNA_34137. Altogether, we speculate that mmu_circRNA_33836, mmu_circRNA_008691, mmu_circRNA_45901 andmmu_circRNA_34137 may play a central role in learning and memory. Importantly, in our previous study, mmu_circRNA_33836 and mmu_circRNA_34137 were down-regulated in the substantia nigra and corpus striatum between Nrf2 (−/−) and Nrf2 (+/+) mice, and this consistent result emphasized the critical function of mmu_circRNA_33836 and mmu_circRNA_34137 in Nrf2-mediated neuroprotection (Yang et al., 2018).

Parkinson’s disease, as the second most prevalent neurodegenerative disorder after AD in the world, is characterized by cardinal motor symptoms, such as bradykinesias, rigidity, postural instability, resting tremor, and non-motor symptoms including psychiatric problems, autonomic disturbances, pain, fatigue, and impaired cognition in executive functioning, memory and spatial behavior during the early stage of the disease (Vingerhoets et al., 2003; Chaudhuri and Odin, 2010). The loss of dopaminergic neurons in the substantia nigra with Lewy bodies (intracytoplasmic inclusion deposits of aggregated alpha-synuclein and ubiquitin protein, and damaged nerve cells) is the primary pathology of PD (Jankovic, 2008; Wakabayashi et al., 2010). Much effort has been made to explore the mechanisms underlying the pathogenesis of PD. Studies of mitophagy in PD support the role of dysfunctional autophagy as a causative factor in neurodegenerative diseases (Menzies et al., 2017). A growing number of studies highlight that disturbance in mTOR signaling in the brain affects multiple pathways including glucose metabolism, energy production, mitochondrial function, cell growth and autophagy, which are key players in age-related cognitive decline (Perluigi et al., 2015). However, a critical question of whether mTOR is neuroprotective or potentially promotes PD pathogenesis has been raised (Blagosklonny, 2008; Lan et al., 2017). In the present study, we also detected that five DEmRNAs, including COX7B2, UBA1Y, VAT1, CYCT and SLC6A3, were enriched in the pathway of Parkinson’s disease. Among them, CYCT and SLC6A3 were co-expressed with the lncRNA ENSMUST00000125413; COX7B2, UBA1Y, and VAT1 were co-expressed with the lncRNAs AK142725 and AK035903, suggesting that these three DElncRNAs (ENSMUST00000125413, AK142725 and AK035903) may play a vital role in neurodegenerative diseases such as PD.

The activity-regulated cytoskeletal-associated protein (Arc) is an immediate early gene that has been broadly involved in hippocampal-dependent learning and memory, and is believed to play an integral role in synapse-specific plasticity (Ploski et al., 2008). Arc mRNA is targeted to activated regions of the dendrite after LTP of the DG, a process dependent on NMDA receptor activation (Moga et al., 2004). According to Plath et al. (2006) despite intact short-term memory, Arc knockout mice failed to form long-lasting memories for implicit and explicit learning tasks and exhibited a biphasic alteration of hippocampal LTP in the DG and area CA1 with an enhanced early and absent late phase. Furthermore, LTD is significantly impaired. They suggested that Arc play a vital role in the consolidation of enduring synaptic plasticity and memory storage (Plath et al., 2006). In the present study, Arc, a significantly up-regulated DEmRNA in the hippocampal tissues of Nrf2 (−/−) mice, was co-expressed with ENSMUST00000125413 and uc008nfy.1, which suggested that these two DElncRNAs were associated with learning and memory.

The cytochrome C oxidase VIIb2 (COX7B2) gene is a member of the cytochrome C oxidase (COX) superfamily (Strausberg et al., 2002). The COX complex, consisting of the COX7B2 subunit with other subunit polypeptides and localized to mitochondria, participates in the electronic transportation process of oxidative phosphorylation (OXPHOS) and in the maintenance of the electrochemical gradient in the mitochondrial membrane (Liang et al., 2004). Oxidative damage is closely associated with the pathogenesis of neurodegenerative diseases including AD, ALS, HD, PD, and stroke (brain ischemia/reperfusion injury) (Kamat et al., 2008). In post mortem brains of advanced PD patients, dysfunction of mitochondrial OXPHOS protein complexes with decreased activity of complex I was detected, which indicates that mitochondrial dysfunction, especially OXPHOS, is strongly implicated in PD (Schapira, 1990; Janetzky et al., 1994; Schapira et al., 2010; Kim-Han et al., 2011). In our results, COX7B2 was co-expressed with AK142725 and AK035903, which suggests that AK142725 and AK035903 may be involved in OXPHOS.

## Conclusion

Compared to Nrf2 (+/+) mice, we identified 1279 DEcircRNAs and 303 DElncRNAs in the hippocampus of Nrf2 (−/−) mice. Among them, mmu_circRNA_33836, mmu_circRNA_008691, mmu_circRNA_45901, and mmu_circRNA_34137 may play a critical role in learning and memory. ENSMUST00000125413, uc008nfy.1, AK142725 and AK035903 may be involved in neurodegenerative diseases by mediating the process of learning and memory and OXPHOS. Our study might represent a new avenue for future investigations to better understand the molecular mechanisms of Nrf2-mediated neuroprotection.

## Data Availability

Publicly available datasets were analyzed in this study. This data can be found here: https://www.ncbi.nlm.nih.gov/geo/query/acc.cgi?acc=GSE122421; https://www.ncbi.nlm.nih.gov/geo/query/acc.cgi?acc=GSE122422.

## Ethics Statement

In this study, the animal experiments complied with the regulations of the Animal Welfare Act of the National Institutes of Health Guide for the Care and Use of Laboratory Animals (NIH Publication No. 85-23, revised 1996) and were approved by the ethics committee of Hebei Medical University (IACUC-Hebmu-Glp-2016017).

## Author Contributions

S-GS and LW contributed to the conception of the study. R-JZ, YL, and QL contributed the materials and performed the experiment. Y-JG, JD, and JM performed the data analyses. R-JZ and YL contributed significantly in writing the manuscript. All authors read and approved the final manuscript.

## Conflict of Interest Statement

The authors declare that the research was conducted in the absence of any commercial or financial relationships that could be construed as a potential conflict of interest.

## Footnotes

1 http://enrich.shbio.com/index/ga.asp

2 http://www.cytoscape.org/

## Supplementary Material

FIGURE S1 The electrophoresis image of the identification of genotypes.

TABLE S1 The primers used in the identification of genotypes.

TABLE S2 Differentially expressed mRNA in the hippocampus between Nrf2 (−/−) and Nrf2 (+/+) mice.

TABLE S3 Differentially expressed circRNAs in the hippocampus between Nrf2 (−/−) and Nrf2 (+/+) mice.

TABLE S4 Differentially expressed lncRNAs in the hippocampus between Nrf2 (−/−) and Nrf2 (+/+) mice.

TABLE S5 The significantly enriched GO and KEGG pathways of DEmRNAs co-expressed with DElncRNAs.
