# Supplementary figures and images for: Differential Expression Profiles and Functional Prediction of Circular RNAs and Long Non-coding RNAs in the Hippocampus of Nrf2-Knockout Mice
Source: Front Mol Neurosci. 2019 Aug 9;12:196. doi: 10.3389/fnmol.2019.00196 (PMC6697070; doi:10.3389/fnmol.2019.00196)

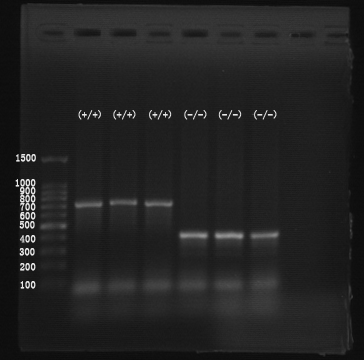

Supplement: FIGURE S1 — The electrophoresis image of the identification of genotypes. [file Image_1.TIF]
