# Supplementary material for: Differential Expression Profiles and Functional Prediction of Circular RNAs and Long Non-coding RNAs in the Hippocampus of Nrf2-Knockout Mice
Source: Front Mol Neurosci. 2019 Aug 9;12:196. doi: 10.3389/fnmol.2019.00196 (PMC6697070; doi:10.3389/fnmol.2019.00196)
Supplement: TABLE S1 — The primers used in the identification of genotypes. [file Table_1.DOCX]

**Table S1 The primers used in the identification of genotypes**

| **Terms** | **Primers** |
| --- | --- |
| Sense for both genotypes | Forward: 5′ TGGACGGGACTATTGAAGGCTG 3′ |
| Antisense for knockout gene | Reverse: 5′ GCGGATTGACCGTAATGGGATAGG 3′ |
| Antisense for Nrf2 | Reverse: 5′ GCCGCCTTTTCAGTAGATGGAGG 3′ |
